# Structure-Based Dissection of the Natural Product Cyclopentapeptide Chitinase Inhibitor Argifin

**DOI:** 10.1016/j.chembiol.2008.02.015

**Published:** 2008-03-21

**Authors:** Ole A. Andersen, Amit Nathubhai, Mark J. Dixon, Ian M. Eggleston, Daan M.F. van Aalten

**Affiliations:** 1 Division of Biological Chemistry & Drug Discovery, College of Life Sciences, University of Dundee, Dundee DD1 5EH, Scotland

**Keywords:** CHEMBIO, PROTEINS

## Abstract

Chitinase inhibitors have chemotherapeutic potential as fungicides, pesticides, and antiasthmatics. Argifin, a natural product cyclopentapeptide, competitively inhibits family 18 chitinases in the nanomolar to micromolar range and shows extensive substrate mimicry. In an attempt to map the active fragments of this large natural product, the cyclopentapeptide was progressively dissected down to four linear peptides and dimethylguanylurea, synthesized using a combination of solution and solid phase peptide synthesis. The peptide fragments inhibit chitinase B1 from *Aspergillus fumigatus* (*Af*ChiB1), the human chitotriosidase, and chitinase activity in lung homogenates from a murine model of chronic asthma, with potencies ranging from high nanomolar to high micromolar inhibition. X-ray crystallographic analysis of the chitinase-inhibitor complexes revealed that the conformations of the linear peptides were remarkably similar to that of the natural product. Strikingly, the dimethylguanylurea fragment, representing only a quarter of the natural product mass, was found to harbor all significant interactions with the protein and binds with unusually high efficiency. The data provide useful information that could lead to the generation of drug-like, natural product-based chitinase inhibitors.

## Introduction

Natural products and compounds directly derived from them continue to play a significant role in modern drug discovery and development (Newman and Cragg, 2007). The most effective compounds targeting chitinases, the enzymes that catalyze the hydrolysis of β(1,4)-linked *N*-acetylglucosamine (chitin), are natural products (reviewed in Andersen et al., 2005). Chitin is an important structural component of the cell wall of fungi (Munro and Gow, 2001), the microfilarial sheath in parasitic nematodes (Harris et al., 2000) as well as the cuticles of insects (Merzendorfer, 2006). Although chitin is absent from mammalian physiology, fungi, nematodes, and insects must all metabolize chitin at important stages of their life cycle; hence, small-molecule chitinase inhibitors have the potential to act as biocontrol agents in both medicine and agriculture. Several natural product chitinase inhibitors have indeed been shown to exhibit interesting biological activities. For example, the pseudotrisaccharide allosamidin, the most extensively studied and generally the most potent chitinase inhibitor, has been shown to inhibit cell separation in fungi (Kuranda and Robbins, 1991; Sakuda et al., 1990), block malaria parasite penetration into the mosquito midgut (Tsai et al., 2001), and inhibit insect larvae development (Sakuda, 1996). Additionally, this compound was shown to decrease the number of inflammatory cells in a mouse model of asthma by targeting the acidic mammalian chitinase (AMCase) (Zhu et al., 2004). Although total syntheses of allosamidin have been reported (Berecibar et al., 1999), the length and complexity of the synthetic routes involved severely limit both the availability of this compound and the scope for structure-based design of novel allosamidin-derived inhibitors.

**Figure 1:**
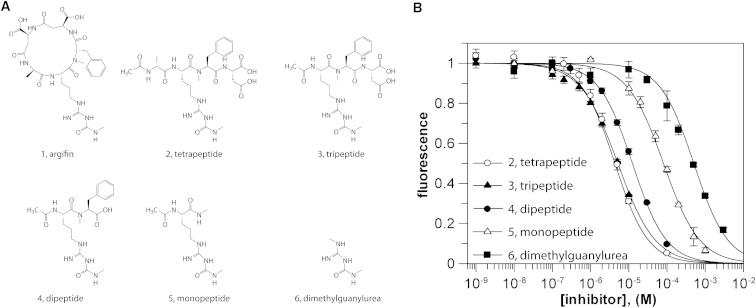
Structures and Activities of Argifin-Derived Peptides (A) Chemical structures. (B) Dose-response curves for the linear peptides against *Af*ChiB1. Chitinase activity represented by relative fluorescence is shown as a function of inhibitor concentration for the argifin-derived peptides. Dose-response curves were fitted with GraFit (Leatherbarrow, 2001) and resulting IC_50_ values are reported in Table 1.

Recently, there has been considerable interest in the ability of peptides to mimic carbohydrate-protein interactions and bind to enzymes and lectins (e.g., Johnson and Pinto, 2002; Vyas et al., 2003; Dharmasena et al., 2007). Synthetic compounds that can mimic such peptide ligands, i.e., peptidomimetics, could provide potential drug candidates with improved stability toward proteolytic breakdown but retaining high potency. Two classes of peptide-based chitinase inhibitors are known: the cyclic proline-containing dipeptides isolated from broth of a marine bacterium (Izumida et al., 1996a) and the cyclic pentapeptides argadin and argifin secreted from fungal strains (Omura et al., 2000; Arai et al., 2000). The synthesis of the natural product cyclo-(L-Arg-D-Pro), or CI-4, is straightforward (Izumida et al., 1996b; Houston et al., 2002a) and in principle further analogs are readily available by substitution of either amino acid residue. However, although several cyclic dipeptide derivatives have been synthesized, their chitinase inhibition is moderate (high micromolar range) (Houston et al., 2002a, 2004). The total syntheses of both argadin and argifin have been achieved (Dixon et al., 2005, 2006) using a combination of solid phase and solution chemistry. For argifin (**1**, Figure 1A), IC_50_ values of 27 nM (Dixon et al., 2005), 33 μM (Houston et al., 2002b), and 4.5 μM (Rao et al., 2005c) have been determined for chitinase B1 from *Aspergillus fumigatus* (*Af*ChiB1), chitinase B from *Serratia marcescens*, and human chitotriosidase (hCHT), respectively. However, although argifin is a potent chitinase inhibitor, its molecular weight and number of hydrogen-bond donors/acceptors well exceed the Lipinski criteria for drug-likeliness (Lipinski, 2000), while its peptide-based framework is also likely to be prone to proteolytic degradation.

Here, we use the available *Af*ChiB1-argifin complex crystal structure to design linearized fragments of this natural product cyclopentapeptide. Using a combination of X-ray crystallographic analysis of the peptide-protein complexes and enzymology, we show that these argifin fragments maintain their binding mode to the enzyme and continue to inhibit potently. Surprisingly, the (tiny) dimethylguanylurea fragment appears to be responsible for forming most of the key interactions and represents an unusually efficient binder and an attractive starting point for structure-based optimization.

## Results and Discussion

### Design and Synthesis of Linear Peptide Inhibitors

**Figure 2:**
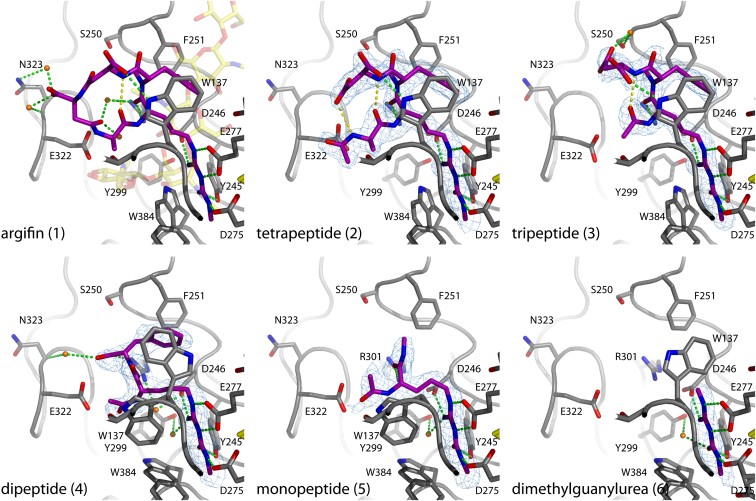
*Af*ChiB1-Inhibitor Complex Crystal Structures Active sites are shown for *Af*ChiB1 in complex with the argifin-derived peptides alongside the published *Af*ChiB1-argifin complex (PDB code: 1W9V; Rao et al., 2005b). The *Af*ChiB1 backbone is shown as a gray ribbon. Side chains lining the inhibitor binding site are shown as sticks with gray carbons and labeled. Dual conformations of Trp137 and Asp175 are shown for the dipeptide and dimethylguanylurea complexes, respectively. The inhibitor molecules are shown as stick models with magenta carbon atoms. Water molecules involved in water-mediated protein-inhibitor interactions are shown as orange spheres. Hydrogen bonds are shown as dotted green lines. Unbiased |F_o_ | − |F_c_ |, ϕ_calc_ electron density maps are shown at 2.5σ for the linear peptide complexes. The position of a chitopentaose oligosaccharide as observed in a previously trapped chitinase-substrate complex (van Aalten et al., 2001) is shown as transparent sticks with yellow carbon atoms, superimposed on the *Af*ChiB1-argifin complex.

The natural product argifin **1** (Figure 1A) is a potent inhibitor of several family 18 chitinases, acting through extensive (peptide) mimicry of the natural carbohydrate substrate (Figure 2; Houston et al., 2002b; Rao et al., 2005b). The structure of the compound in complex with chitinase B from *A. fumigatus* (Rao et al., 2005b; Figure 2) was used as a basis to design a range of peptide fragments that all contain the dimethylguanylurea group previously shown to deeply penetrate the active site pocket (Houston et al., 2002b; Rao et al., 2005b; Figure 2). Hence, the linear tetra-, tri-, and dipeptides **2–4** were generated (Figure 1), along with an isolated Arg derivative **5** with an *N*-methylcarbamoyl-derivatized side chain, and the dimethylguanylurea **6** itself (Figure 1).

For the preparation of **2–4**, Arg-containing di-, tri-, and tetrapeptide precursors were assembled by manual Fmoc SPPS on 2-chlorotrityl chloride polystyrene resin. After cleavage from the resin and side chain deprotection, the Arg(MC) modification was introduced by acylation with MeNHCO_2_Su/DBU/DMF at 40°C, as described for the synthesis of argifin. **2–4** were isolated by preparative HPLC and were fully characterized by NMR and high resolution mass spectrometry. **5** was prepared from Fmoc-L-Arg(Pmc)-OH by standard solution chemistry, but using methyl isocyanate to install the Arg(MC) modification, and was isolated by preparative HPLC and characterized as previously. **6** was prepared from methylguanidine hydrochloride by neutralization with ion exchange resin (Dowex hydroxide form), followed by treatment with methyl isocyanate and isolation as for **5**. Full details of the syntheses are available in the Supplemental Data available with this article online.

### Investigation of Inhibition and Binding Mode of the Argifin Peptide Fragments

Argifin has been shown to be a competitive inhibitor of family 18 chitinases, binding with affinities from in the nanomolar to micromolar range (Arai et al., 2000; Houston et al., 2002b; Rao et al., 2005b). To compare the linear peptides synthesized in this study, inhibition was tested against a fungal chitinase (*Af*ChiB1), the human chitotriosidase (hCHT), and total chitinase activity from a lung homogenate (LH) from a mouse model of chronic asthma. This lung homogenate has previously been shown to mainly contain the acidic mammalian chitinase (AMCase) that has recently been proposed as an antiasthma drug target (McMillan and Lloyd, 2004; Zhu et al., 2004; Schüttelkopf et al., 2006; Table 1). In parallel, *Af*ChiB1 crystals were soaked in solutions containing compounds **2–6** and the resulting crystal structures investigated. The five structures were solved to a maximum resolution of 1.90–2.2 Å (Table 2), similar to the published (2.0 Å) *Af*ChiB1-argifin complex, and thus the structures possess a comparable amount of solvent detail. The data yielded electron density maps showing clear positions for the ligand, confirming the structure and stereochemistry of the synthesized linear peptides (Figure 2).

Argifin itself binds to family 18 chitinases through subsites (i.e., where the individual sugars of the chitin polymer are known to bind) −1, +1, and +2 of the active site, following standard glycoside hydrolase subsite nomenclature (Davies et al., 1997), with hydrolysis taking place between the −1 and +1 sugar. In the previously published *Af*ChiB1-argifin crystal structure (Figure 2), all inhibitor peptide bonds are in the *trans* configuration except for the *cis* L-Arg to *N*-MePhe bond (Rao et al., 2005b). The *N*-methyl carbamoyl nitrogen hydrogen bonds to the side chain of Asp175, whereas its oxygen forms a hydrogen bond to the hydroxyl group of Tyr245. The arginine guanidinium group forms two hydrogen bonds with Glu177 (the catalytic acid) and one with the side chain of Asp246. Additionally, the first *iso*Asp side chain generates a hydrogen bond with the Trp137 side chain, and the argifin arginine carbonyl forms two hydrogen bonds with the Arg301 side chain. One intramolecular hydrogen bond is observed between the first *iso*Asp backbone N and the D-Ala carbonyl O, associated with a β-turn centered on the L-Arg-L-MePhe dipeptide moiety (Figure 2). Numerous water-mediated hydrogen bonds are observed involving Arg301, Glu322, and Asn323. The side chains of Tyr48, Met243, and Trp384 generate hydrophobic interactions and surround the *N*-methyl carbamoyl group, whereas the side chains of Trp137 and Phe251 form extensive stacking interactions with the argifin L-Phe. Almost all of these residues are highly conserved in family 18 chitinases, explaining the potency of this inhibitor reported for several enzymes.

### Argifin Linearization and Truncation Generates an Active Tetrapeptide

Linearization of the cyclopentapeptide argifin **1** by removal of one of the Asp residues results in tetrapeptide **2** and surprisingly leads to retention of significant inhibitory activity (IC_50_ = 4.3 μM against *Af*ChiB, Table 1). In agreement with this, the crystal structure (Figure 2) reveals that it binds the chitinase with the Arg(MC) side chain in an identical position to that observed in the *Af*ChiB1-argifin complex, with a similar conformation of the peptide backbone (shifts up to 0.6 Å for equivalent argifin backbone atoms). Hence, the inhibitor fills the −1, +1, and +2 subsites, and all direct hydrogen bonds and intramolecular hydrogen bonds observed in the argifin complex are conserved (Figure 2). The only significant differences are the loss of water-mediated hydrogen bonds to Glu322/Asn323 and a *cis* amide bond in the N-terminal *N*-acetyl group, allowing an additional internal hydrogen bond between the C-terminal carboxylate and the backbone nitrogen of D-Ala (Figure 2). The total surface area buried by argifin and **2** are identical (141 Å^2^). Thus, it seems likely that removal of the second *iso*Asp and linearization of the natural product lowers the affinity of binding to the chitinases through entropic penalties incurred upon complexation of the more flexible, acyclic structure of **2** and/or loss of several water-mediated hydrogen bonds.

### Further Truncation of the Tetrapeptide Generates an Active Tripeptide

It is apparent from the *Af*ChiB-argifin/tetrapeptide complexes that the D-Ala side chain does not make significant interactions with the enzyme active site (Figure 2). Indeed, the tripeptide **3**, lacking the D-Ala residue, inhibits chitinase activity of *Af*ChiB1, hCHT, and LH with IC_50_ values similar to those of the tetrapeptide **2** (Figure 1B; Table 1). Again, crystallographic analysis of a complex with *Af*ChiB (Figure 2) shows that the Arg(MC) side chain binds in its usual position and that the tripeptide backbone adopts a conformation almost identical to that observed in the *Af*ChiB1-argifin complex (shifts up to 0.5 Å for equivalent argifin backbone atoms). The differences from the tetrapeptide are the displacement of the *iso*Asp C terminus, the loss of the extra internal hydrogen bond, and an extra water-mediated hydrogen bond with Ser250.

### An Argifin Dipeptide Fragment Showing Micromolar Inhibition

To further narrow down the pharmacophore of the argifin natural product, the tripeptide-protein interactions of **3** were evaluated for the individual amino acids to design the most potent dipeptide. Both the derivatized argifin and the *N*-methylated phenylalanine make tight interactions with the active site (Figure 2) and truncation of these amino acids was expected to significantly compromise binding. The only interaction the remaining *iso*Asp side chain makes with the protein is to accept a hydrogen bond from the Trp137 indole, although this interaction is fully conserved in the argifin, tetrapeptide and tripeptide complexes, and mutation of the tryptophan is known to completely abolish argifin inhibition (Rao et al., 2005b; Figure 2). Nevertheless, it was decided to synthesize dipeptide **4**, which inhibits the chitinases with IC_50_ values less than an order of magnitude higher than those measured for the tri/tetrapeptides **2** and **3** (Figure 1B; Table 1). The *Af*ChiB1-dipeptide complex (Figure 2) reveals that the terminal Arg(MC) moiety generates the same five hydrogen bonds with Asp175, Glu177, Tyr245, and Asp246 as seen in the *Af*ChiB1 complexes with argifin. However, the loss of an internal hydrogen bond and the hydrogen bond with the side chain of Trp137 appears to induce significant conformational changes in the backbone (shifts up to 3.4 Å for equivalent argifin backbone atoms), with the L-Arg-*N*-methyl-L-Phe peptide bond adopting a *trans* configuration (Figure 2). Consequently, the Arg301 to L-Arg carbonyl hydrogen bond is replaced with a hydrogen bond from Arg301 to the MePhe carboxylate, and the MePhe side chain is displaced to only partially occupy the +2 subsite through a reduced interaction with Phe251 (Figure 2). The shift of the inhibitor backbone also causes Trp137 to settle in a dual conformation, with an additional conformation pointing toward the indole group of Trp384, which itself is also displaced. However, these conformational changes also result in the generation of three new water-mediated hydrogen bonds that may partially compensate for the loss of direct hydrogen bonds. In addition, the considerably smaller dipeptide generates a protein contact surface of equivalent size to that observed in the *Af*ChiB1-tripeptide complex (Table 2).

### The *N*-Methylcarbamoylated Arginine Is the Pharmacophoric Argifin Amino Acid

Despite the linearization and truncation of argifin down to a dipeptide, significant activity is retained and the pharmacophore is thus likely to be an even smaller fragment. We next investigated the activity of the acetylated single amino acid, “monopeptide” **5**. Strikingly, this monopeptide still inhibits *Af*ChiB1 with an IC_50_ value of 81 μM (Figure 2; Table 1), although this is 3 orders of magnitude higher than inhibition by the intact cyclopentapeptide **1** and 40-fold higher than the linear tetrapeptide **2**. The crystal structure of the *Af*ChiB-monopeptide complex shows that despite the removal of the MePhe residue and concomitant loss of the hydrophobic interactions with Trp137/Phe 251, the modified arginine side chain still establishes the same five hydrogen bonds as observed in the argifin complex (Figure 2). Similar to the dipeptide complex, Trp137 is flipped with a 120° rotation around the Cα-Cβ bond, allowing stacking interactions with the L-Arg backbone and the N-terminal acetyl group.

### Dimethylguanylurea Is an Active Chitinase Inhibitor Fragment

Because the terminal atoms of the modified arginine residue of **1–5** appear to make the majority of the interactions with the protein, we decided to investigate inhibition of the dimethylguanylurea **6** itself. Strikingly, this small fragment, consisting of only 9 heavy atoms and with a molecular weight of 130 Da, still shows detectable inhibition of *Af*ChiB1 (IC_50_ = 500 μM) and other chitinases (Figure 1B; Table 1). In the dimethyl-guanylurea complex crystal structure with *Af*ChiB1, the fragment is well ordered, occupies the same position as observed in the argifin and linear peptide complexes, and is able to make five hydrogen bonds (Figure 2). The reduction in potency compared to the monopeptide can be explained by the loss of the L-Arg carbonyl to Arg301 hydrogen bond as well as a considerably smaller buried surface area (Table 2) due to loss of interactions with Trp137 (Figure 2).

### Concluding Remarks

In order to establish the minimum elements of the argifin structure that are required for activity and provide the basis for the design of new inhibitors derived from this lead, five acyclic fragments of the natural product have been synthesized, inspired by the previously published structures of argifin-chitinase complexes (Houston et al., 2002b; Rao et al., 2005b). The mode of binding of these fragments with *Af*ChiB has been elucidated and allowed differences in inhibitor potency observed upon incremental structural simplification to be explained. Although such analyses of potency changes upon systematic deconstruction of optimized synthetic inhibitors have previously been reported (Hajduk, 2006), to the best of our knowledge this is the first example of a combined kinetic and crystallographic investigation of the binding of a systematically dissected natural product.

The argifin peptide fragments can be ranked tetrapeptide > tripeptide > dipeptide > monopeptide > dimethylguanylurea with respect to their potencies (Table 1). This rank can be explained by the progressive loss of protein-inhibitor contacts observed in the crystal structures of the complexes in conjunction with predicted entropic penalties linked to linearization of a conformationally restricted cyclic peptide. However, all peptide fragments, including the minimal dimethylguanylurea, maintain a core minimum of interactions, namely the hydrogen bonds to Asp175, Glu177 (the catalytic acid), Tyr245, and Asp246 and stacking with Trp384. With the exception of Asp246, these residues are conserved in family 18 chitinases throughout all kingdoms of life. The ranking of the argifin fragments is also in good agreement with a previously published mutagenesis study to identify key *Af*ChiB-argifin interactions, which showed large effects on argifin affinity if amino acids either hydrogen bonding to the modified argifin side chain or showing stacking interactions were mutated (Rao et al., 2005b). Other known family 18 chitinase inhibitors, such as allosamidin, diketopiperazines, and purine derivatives, also derive their potencies from interacting with these active site residues (Terwisscha van Scheltinga et al., 1994; Houston et al., 2002a; Rao et al., 2005a).

The surprising result of this study is that the pharmacophore of the large cyclopentapeptide argifin **1** is a 9 atom fragment, the dimethylguanylurea **6**. In absolute terms, this is a poor inhibitor, with an IC_50_ of 500 μM compared to 27 nM for the natural product (Table 1). However, viewing the same data in terms of ligand efficiency (Kuntz et al., 1999; Hopkins et al., 2004; Abad-Zapatero and Metz, 2005), here defined as binding efficiency index (BEI) (Abad-Zapatero and Metz, 2005), dimethylguanylurea is the most efficient argifin fragment with a BEI of 27.7, a significantly more efficient binder than argifin itself (BEI = 11.7 with a molecular weight of 676 Da). As evidenced by the structures of argifin-chitinase complexes (Houston et al., 2002b; Rao et al., 2005b; Figure 2), there are relatively large complementary buried surfaces on the inhibitor and protein, suggesting that while not very efficient, argifin may be a very selective inhibitor. Conversely, it is likely that the small dimethylguanylurea fragment with its limited contacts will also fit a number of other active sites.

In a given series, inhibitor potency commonly correlates with molecular weight, and hence ligand efficiency is a more useful measure than potency when selecting a lead compound for further optimization into a drug or monitoring progress in a drug development program. Recently, a retrospective analysis of several drug discovery campaigns suggested that selecting relatively small, efficient binders as a starting framework increases the chances of obtaining a final optimized ligand with high potency that does not violate the Lipinski rule-of-five (Hajduk, 2006; Lipinski, 2000) and fits the restrictions for acceptable absorption and permeability properties. The dimethylguanylurea fits all Lipinski rules, with 4 hydrogen bond donors, 1 hydrogen bond acceptor, a molecular mass of 130 Da, and clogP = −0.48. In addition, removal of all peptide bonds eliminates argifin's intrinsic susceptibility to proteolytic breakdown and is thus likely to give more favorable pharmacokinetic properties. Using the observation that, in an ideal optimization program, ligand efficiency remains constant throughout lead optimization (Hopkins et al., 2004; Hajduk, 2006), dimethylguanylurea serves as an attractive fragment lead with a speculative potential to be optimized to a compound with a K_i_ of 60 nM at a molecule weight of 260 Da or a K_i_ of 16 pM at a weight of 390 Da.

## Significance

**Natural products are often large, non-drug-like molecules requiring lengthy routes toward total synthesis, yet provide useful guides for the medicinal chemist and mechanistic enzymologist alike. Argifin, a large natural product cyclopentapeptide, inhibits family 18 glycoside hydrolases (chitinases) by extensive mimicry of protein-substrate (carbohydrate) interactions, as shown by previous structural/mutagenesis studies. This work shows, by cumulative truncation of the cyclic peptide, that the majority of this 620 Da natural product is not required for efficient inhibition. Strikingly, most of the activity of the peptide resides in a 130 Da dimethylguanylurea that binds deep in the active site and retains micromolar inhibition. The data suggest that by rational decoration of this natural product-derived fragment, more potent, and drug-like, chitinase inhibitors can be obtained.**

## Experimental Procedures

### Expression, Purification, and Crystallization of *Af*ChiB1

*A. fumigatus* chitinase B1 (*Af*ChiB1) was overexpressed in *Escherichia coli* and purified as previously described (Rao et al., 2005b). Pure enzyme was spin concentrated to 27 mg/ml in 25 mM Tris-HCl (pH 8). The protein was crystallized from 1.2 M Li_2_SO_4_, 0.1 M Tris-HCl (pH 9) using the hanging drop method. Crystals used for soaking were washed three times in 0.1 M sodium citrate (pH 5.5) and 1.4 M Li_2_SO_4_, with the final drop containing 1 mM inhibitor, using 2 hr of soaking time. Crystals were cryoprotected in 3 M Li_2_SO_4_ and subsequently flash frozen in liquid nitrogen.

### Data Collection and Structural Determination of Binary Chitinase-Peptide Complexes

X-ray diffraction data for the tetrapeptide, tripeptide, dipeptide, and monopeptide complexes were collected at ID14-EH2 at the European Synchrotron Radiation Facilities (ESRF). X-ray diffraction data for the dimethylguanylurea complex were collected using a rotating anode. All data sets were collected at 100 K. Processing and scaling were done using the HKL suite of programs (Otwinowski and Minor, 1997). Cross-validation was applied by excluding 1% of the reflections throughout the refinement procedure. Rigid body and simulated annealing followed by several rounds of combined refinement (energy minimization and B-factor refinement) were performed using CNS (Brunger et al., 1998). O (Jones et al., 1991) was used for manual adjustments of the structures, and water molecules were included as oxygen atoms after each round of combined refinement using appropriate criteria. Topologies for the linear peptides were obtained using the PRODRG server (Schüttelkopf and van Aalten, 2004) and the ligands were only included when fully defined by unbiased |F_o_ | − |F_c_ |, ϕ_calc_ electron density maps (Figure 2). The final models include two monomers in the asymmetric unit. In the interest of simplicity, the structures are discussed consistently using the first monomer of the coordinate files unless otherwise stated.

### *Af*ChiB1, hAMCase, and Lung Homogenate Enzymology

Chitinase activities for *Af*ChiB1 (Rao et al., 2005b), hCHT (Boot et al., 1998), and total chitinase activity in lung homogenate samples from a mouse model of chronic asthma were determined as previously described (Schüttelkopf et al., 2006). Activities were measured in a final volume of 50 μl, and IC_50_ determinations were done in the presence of different concentrations of inhibitor. *Af*ChiB1 (2 nM) was incubated with 20 μM 4-methylumbelliferyl-β-D-*N-N'*-diacetylchitobiose (4MU-GlcNAc_2_; Sigma) and 0.25 mg/ml bovine serum albumin in 100 mM citric acid, 200 mM Na_2_HPO_4_ (pH 5.5). hCHT (0.3 nM) was incubated with 22 μM 4-methylumbelliferyl-β-D-*N-N'*-triacetylchitobiose (4MU-GlcNAc_3_; Sigma) and 0.25 mg/ml bovine serum albumin in 100 mM citric acid, 200 mM Na_2_HPO_4_ (pH 5.2). Lung homogenate (39 μg/ml) (obtained as described previously [Schüttelkopf et al., 2006]) was incubated with 20 μM 4MU-GlcNAc_2_ in 100 mM citric acid, 200 mM Na_2_HPO_4_ (pH 5.5). All reactions were run for 10 min at 37°C, and liberated 4-methylumbelliferone (4MU) was quantified after addition of 25 μl 3 M glycine-NaOH (pH 10.6) using an Flx 800 microtiterplate fluorescence reader (Bio-Tek instruments) with 40 nm slits and excitation and emission wavelengths of 360 nm and 460 nm, respectively. All experiments were performed in triplicate, and production of 4MU was linear for the incubation period used with less than 10% of available substrate hydrolyzed.

## Figures and Tables

**Table 1:** Chitinase Inhibition of Argifin-Derived Peptides

	*Af*ChiB1 IC_50_	*Af*ChiB1 BEI	hCHT IC_50_	LH IC_50_
Argifin	0.027	11.7	4.5	0.030−0.003
Tetrapeptide	4.3±0.2	9.1	28−2	2.9−0.2
Tripeptide	5.1±0.2	10.2	68−4	8.2−0.5
Dipeptide	12±1	12.0	190−30	27−2
Monopeptide	81±4	15.3	1000−100	180−20
Dimethylguanylurea	500±20	27.7	5800−400	1030−40

IC_50_ values of argifin and the peptide derivatives against *Af*ChiB1, hCHT, and mouse lung homogenate (LH) are given in micromolar. Argifin inhibition of *Af*ChiB1 and hCHT have been reported previously (Rao et al., 2005b; Dixon et al., 2005). For *Af*ChiB1, ligand efficiency is expressed as the Binding Efficiency Index (Abad-Zapatero and Metz, 2005), BEI = −log(K_i_)/M, with M being the mass in kilodaltons and K_i_s derived from the IC_50_ data using the Cheng and Prusoff equation.

**Table 2:** Summary of Data Collection, Structure Refinement, and Analysis

	Tetrapeptide	Tripeptide	Dipeptide	Monopeptide	Dimethylguanylurea
Resolution (Å)	20−1.95 (2.02−1.95)	20−2.05 (2.12−2.05)	20−2.00 (2.07−2.00)	20−1.90 (1.97−1.90)	20−2.20 (2.28−2.20)
Unit cell (Å)	a = b = 117.06	a = b = 117.42	a = b = 117.07	A = b = 117.18	a = b = 117.77
	c = 99.75	c = 99.54	c = 99.95	C = 100.05	c = 99.57
# Unique reflections	95975 (9051)	84294 (8096)	89613 (8652)	105305 (10382)	67434 (6511)
Multiplicity	3.7 (3.2)	3.7 (2.8)	3.6 (3.3)	3.3 (3.2)	3.2 (2.8)
R_merge_ (%)	9.2 (42.7)	11.3 (37.7)	10.6 (49.1)	8.4 (42.0)	7.9 (49.2)
I/σI	17.2 (2.5)	14.3 (2.7)	16.2 (2.5)	17.5 (2.5)	15.2 (2.1)
Completeness (%)	98.0 (92.7)	99.6 (95.8)	98.6 (96.0)	99.1 (98.3)	97.9 (95.6)
# atoms in refinement	7141	7189	7100	7238	6776
# solvent molecules	842	910	827	1002	552
R, R_free_ (%)	17.6, 19.7	17.2, 19.5	17.7, 21.5	16.6, 18.7	18.7, 22.0
<B > protein (Å^2^)	24	20	26	23	30
<B > ligand (Å^2^)	41	46	49	31	50
R.m.s.d. from ideal geometry					
Bond lengths (Å)	0.011	0.011	0.011	0.012	0.011
Bond angles (°)	1.53	1.53	1.57	1.54	1.54
Inhibitor-buried area (Å^2^)	141	127	123	97	59

Values for the highest resolution shell are given in brackets.

## Accession Numbers

The coordinates and structure factors have been deposited with the PDB entries 3CH9, 3CHC, 3CHD, 3CHE, and 3CHF.

## Supplemental Data

Document S1. Supplemental Experimental Procedures
