# Supplementary material for: Structure-Based Dissection of the Natural Product Cyclopentapeptide Chitinase Inhibitor Argifin
Source: Chem Biol. 2008 Mar 21;15(3):295–301. doi: 10.1016/j.chembiol.2008.02.015 (PMC3764403; doi:10.1016/j.chembiol.2008.02.015)
Supplement: Document S1. Supplemental Experimental Procedures [file mmc1.pdf]

Chemistry & Biology *15*

Supplemental Data

Structure-Based Dissection of the Natural Product Cyclopentapeptide Chitinase Inhibitor Argifin

Ole A. Andersen, Amit Nathubhai, Mark J. Dixon, Ian M. Eggleston, and Daan M. F. van Aalten

Supplemental Experimental Procedures

General

Chemical reagents were purchased from Sigma, Aldrich, Fluka, Acros, Lancaster and Novabiochem. Anhydrous CH_2Cl_2 was obtained by distillation over calcium hydride, anhydrous THF and Et_2O over sodium/benzophenone. All other solvents were purchased from Fisher Scientific. Analytical TLC was performed using silica gel 60 F_{254} pre-coated on aluminium sheets (0.25 mm thickness). Flash chromatography was performed on silica gel 60 (35-70 micron) from Fisher Scientific. ^{1}H and ^{13}C NMR were recorded using Bruker Avance 500MHz, JEOL JMN GX-270MHz or EX-400MHz spectrometers. *J* values are given in Hz. Analytical RP-HPLC was performed on a Dionex HPLC system equipped with a Dionex Acclain 3 µm C-18 (150 x 4.6mm) column with a flow rate of 1 mL/min. Semi-preparative RP-HPLC was performed on a Dionex HPLC system equipped with a Phenomenex Gemini 5µm C-18 (250 x 10mm) column with a flow rate of 2.5 mL/min, or a Phenomenex Gemini 5µm C-18 (250 x 30mm) column with a flow rate of 22.5 mL/min. Mobile phase A was 0.1% TFA in water, mobile phase B was 0.1% TFA in acetonitrile. Gradient 1 was *T* = 0 min, *B* = 5%; *T* = 10 min, *B* = 95%; *T* = 15 min, *B* = 95%; *T* = 15.1 min, B = 5%; *T* = 18.1 min, *B* = 5%. Gradient 2 was *T* = 0 min, *B* = 5%; *T* = 20 min, *B* = 40%; *T* = 22 min, *B* = 95%; *T* = 27 min, *B* = 95%; *B*= 5%; *T* = 32 min, *B* = 5%. Gradient 3 was *T* = 0 min, *B* = 5%; *T* = 15 min, *B* = 32.5%; *T* = 17, *B* = 95%; *T* = 22 min, *B* = 95%; *T* = 23 min, *B* = 5%; *T* = 30 min, *B* = 5%. Gradient 4 was *T* = 0 min, *B* = 5%; *T* = 20 min, *B* = 60%; *T* = 22 min, *B* = 95%; *T* = 27 min, *B* = 95%; *T* = 27.1%, *B* = 5%; *T* = 36 min, *B* = 5%. Gradient 5 was *T* = 0 min, *B* = 5%; *T* = 12 min, *B* = 7.8%; *T* = 14 min, *B* = 95%; *T* = 19 min, *B* = 95%; *T* = 21 min, *B* = 5%, *T* = 29 min, *B* = 5%; *T* = 29.1 min, *B* = 5%. Gradient 6 was *T* = 0 min, *B* = 5%; *T* = 30 min, *B*

= 12%; T = 32 min, B% = 95%; T = 37 min, B = 95 %; T = 38 min, B = 5%; T = 42 min, B = 5%; T = 42.1 min, B = 5%.

High resolution mass spectrometry was performed using a Bruker MicroTOF autospec electrospray ionisation mass spectrometer.

Ac-D-Ala-L-Arg(MC)-L-MePhe-*iso*-L-Asp-OH (Tetrapeptide)

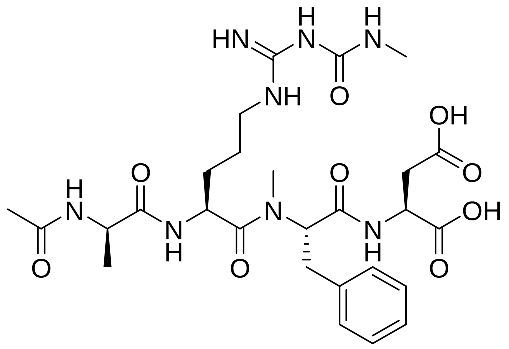

Solid Phase Synthesis of Ac-D-Ala-L-Arg-L-MePhe-iso-L-Asp-OH:

Synthesis was performed manually on 2-chlorotrityl polystyrene resin (0.30 g, 0.42 mmol). Loading of the resin was achieved by treating the resin with a solution of Fmoc-Asp-OBu^t (0.35 g, 0.84 mmol) and DIPEA (0.29 μL, 1.68 mmol) in CH_2Cl_2 for 90 min. Fmoc deprotection was achieved by treatment with piperidine/DMF (v/v, 1:4) for 4 x 3 min. Peptide couplings were performed using Fmoc-amino acid (2 equiv), PyBOP (1.9 equiv), HOBt (2 equiv) and DIPEA (4 equiv) in CH_2Cl_2/DMF (v/v 3:1) for 90 min, except for the coupling to MePhe, where Fmoc-Arg(Pmc)-OH (2 equiv), PyBrOP (2 equiv) and DIPEA (4 equiv) in CH_2Cl_2/DMF (v/v 3:1) for 3 x 90min were used. Solid-phase reactions were monitored by use of a qualitative Kaiser test for the detection of primary amines and the chloranil test for detection of secondary amines. After coupling of Fmoc-Arg(Pmc)-OH, the resin was divided into two equal portions, which were then used for the preparation of **(tetrapeptide)** and **(tripeptide)** respectively. For **(tetrapeptide)**, after coupling of Fmoc-D-Ala-OH, the resin-bound tetrapeptide was N(α)-deprotected and acetylated by treatment with acetic anhydride/ CH_2Cl_2/pyridine (v/v/v 0.5:1:8.5) for 35 min. Cleavage from the resin was then effected by treatment with TFA/thioanisole/CH_2Cl_2/H_2O (16:2:1:1, 5mL). The cleavage solution was concentrated *in vacuo*, and the residue was dissolved in H_2O (7 mL) and washed with CH_2Cl_2 (6 x 7mL). The aqueous extract was lyophilised to give

the fully deprotected Arg-containing tetrapeptide intermediate as the trifluoroacetate salt as a white solid (12.3 mg, 0.018 mmol).

Acylation:

A stirred solution of the crude peptide from above in DMF (2 mL) was treated with *N*-succinimidyl-*N*-methyl carbamate (24.9 mg, 0.145 mmol) and DBU (14.5 µL, 0.096 mmol) at 40 °C for 36h. The reaction mixture was concentrated *in vacuo* and the crude product was purified by RP semi-preparative HPLC (gradient 2) to give **(tetrapeptide)** as the trifluoroacetate salt a white solid (4.0 mg, 26% yield, based on 0.21 mmol starting resin). RP-HPLC (analytical, gradient 1, λ = 214 nm): t_R = 9.4 min. HRMS(ES+) found 621.3008 $[M+H]^+$, $C_{27}H_{41}N_8O_9$ requires 621.2991.

Ac-L-Arg(MC)-L-MePhe-*iso*-L-Asp-OH (Tripeptide)

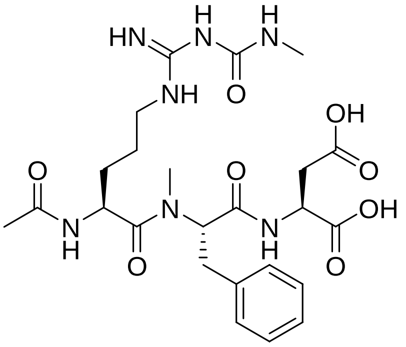

Solid Phase Synthesis of Ac-L-Arg-L-MePhe-iso-L-Asp-OH:

The resin-bound tripeptide (see above) was N(α)-deprotected and acetylated as for **(tetrapeptide**). Following cleavage from the resin and isolation as before, the fully deprotected Arg-containing tripeptide intermediate was obtained as the trifluoroacetate salt as a white solid (10.9 mg, 0.017 mmol).

Acylation:

A stirred solution of the crude peptide from above in DMF (2 mL) was treated with *N*-succinimidyl-*N*-methyl carbamate (23.2 mg, 0.135 mmol) and DBU (13.6 µL, 0.091 mmol) at 40 °C for 36 h. The reaction mixture was concentrated *in vacuo* and the crude product was purified by RP semi-preparative HPLC (gradient 2) to give **(tripeptide)** as the trifluoroacetate salt a white solid (1.9 mg, 14% yield, based on

0.21 mmol starting resin). RP-HPLC (analytical, gradient 1, λ = 214 nm): t_R = 9.4 min. HRMS(ES+) found 550.2608 $[M+H]^+$, $C_{24}H_{36}N_7O_8$ requires 550.2620.

Ac-L-Arg(MC)-L-MePhe (Dipeptide)

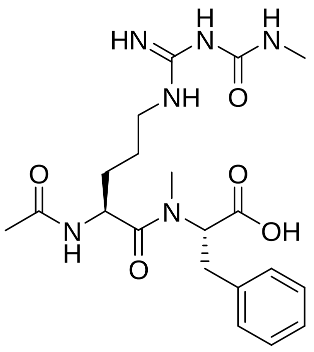

Solid Phase Synthesis of Ac-L-Arg-L-MePhe-iso-L-Asp-OH:

This was performed as for **(tetrapeptide)**, using 2-chlorotrityl polystyrene resin (0.20 g, 0.28 mmol). After cleavage from the resin and isolation as before, the fully deprotected Arg-containing dipeptide intermediate was obtained as the trifluoroacetate salt as a white solid (28.2 mg, 0.057 mmol).

Acylation:

A stirred solution of the crude peptide from above in DMF (2 mL) was treated with *N*-succinimidyl-*N*-methyl carbamate (88.3 mg, 0.513 mmol) and DBU (102 μL, 0.684 mmol) at 40 °C for 36h. The reaction mixture was concentrated *in vacuo* and the crude product was purified by RP semi-preparative HPLC (gradient 2) to give **(dipeptide)** as the trifluoroacetate salt as a white solid (4.5 mg, 7% yield). RP-HPLC (analytical, gradient 1, λ = 214 nm): t_R = 9.7 min. HRMS(ES+) found 435.2334 $[M+H]^+$, $C_{20}H_{31}N_6O_5$ requires 435.2350.

Ac-L-Arg(MC)-NHMe (xxxxx)

Fmoc-L-Arg(Pmc)-NHCH$_3$

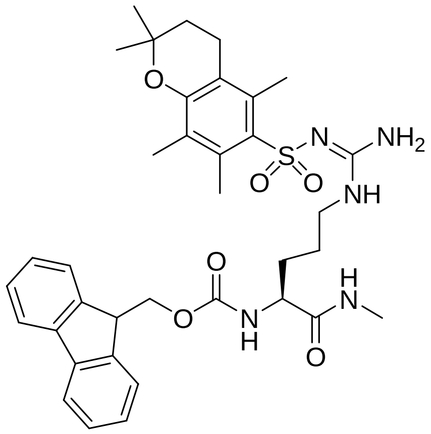

A stirred solution of Fmoc-L-Arg (Pmc)-OH (0.50 g, 0.75 mmol) in 1:1 CH_2Cl_2:DMF (6 mL) at 0 °C, was treated with HOBt.H_2O (0.14 g, 1.06 mmol) and EDC.HCl (0.15 g, 0.75 mmol). The reaction mixture was stirred for 1 h, then methylamine hydrochloride (51 mg, 0.75 mmol) and DIPEA (0.29 mL, 1.66 mmol) were added, and the mixture was allowed to warm to room temperature overnight. The solvent was evaporated and the residue was dissolved in EtOAc (15 mL) and washed with 5% citric acid (2 x 8 mL), 5% $NaHCO_3$ (2 x 8 mL), water (2 x 8 mL) and brine (8 mL). The organic extract was dried ($MgSO_4$) and the solvent was evaporated. The crude product was purified by flash chromatography (CH_2Cl_2 to 9:1 CH_2Cl_2/MeOH) to give a colourless oil (0.41g, 80%). R_f 0.31 (CH_2Cl_2/MeOH, (9:1); δ_H (270 MHz, $CDCl_3$): 1.24 (6H, s, 2 x CH_3) 1.47-1.84 (6H, m, β CH_2 + γ CH_2 + C*H$_2$*C(CH_3)$_2$) 2.05 (3H, s, ArCH_3) 2.43-2.61 (8H, m, 2 x CH_3, ArCH_2) 2.69, 2.71 (1.5H each, s, NHCH_3 rotamers) 3.10-3.39 (2H, m, δ-CH_2) 4.03 (1H, t, *J*=7.1 α CH) 4.18-4.37 (3H, m, (Fmoc CH + CH_2) 6.19-6.37 (3H, m, (NH_2 + N*H*CH_3) 7.15 -7.24 (3H, m, CO_2NH + 2 x Ar CH) 7.29 (2H, t, *J*=7.3, 2 x ArCH) 7.49 (2H, d, *J*=7.4, 2 x ArCH) 7.67 (2H, d, *J*=7.5, 2 x ArCH); δ_C (101 MHz, $CDCl_3$): 12.26 (ArCH_3), 17.64 (ArCH_3), 18.69 (ArCH_3), 21.50 (CH_2), 25.75 (*C*H_2), 26.35 (CH_3)$_2$), 26.82 ((CH_3)$_2$), 30.02 (CH_2), 32.80 ((CH_3)$_2$C*C*H$_2$CH$_2$), 40.04 (δ-CH_2), 47.01 (NHCH_3), 53.57 (CH) 54.57 (CH), 67.05 (CH_2O), 73.79 (C=N), 118.15 (*C*(CH_3), 120.01 (*C*-CH_2 Pmc), 124.29 (2 x ArCH), 125.23 (2 x ArCH), 127.13 (2 x ArCH), 127.78 (2 x ArCH), 133.00 (C-S), 134.91 (*C*(CH_3), 135.50. (*C*(CH_3), 141.27 (2 x ArC), 143.52 (2 x ArC), 153.40

(CO_2NH), 156.29 (Fmoc CO), 173.21 (CONH(CH_3). HRMS(ES+) found 676.3179 $[M+H]^+$, $C_{36}H_{46}N_5O_6S$ requires 676.3163.

Ac-L-Arg(Pmc)-NHCH$_3$

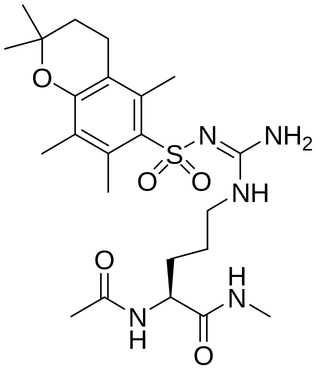

A stirred solution of Fmoc-L-Arg(Pmc)-$NHCH_3$ (0.20 g, 0.29 mmol) in DMF (6 mL) was treated with diethylamine (0.47 mL, 4.56 mmol). After 45 min, the solvent was evaporated and the residue was dissolved in CH_2Cl_2 (6 mL), cooled to 0°C, and the resulting solution treated with acetic anhydride (86 μL, 0.91 mmol), DMAP (36 mg, 0.29 mmol), and DIPEA (95 μL, 0.55 mmol). The reaction mixture was allowed to warm to room temperature and was stirred overnight, then it was diluted with CH_2Cl_2 (10 mL) and was washed with 5% citric acid (2 x 8 mL), 5% $NaHCO_3$ (2 x 8 mL), water (2 x 8 mL) and brine (8 mL). The organic extracts were dried ($MgSO_4$) and the solvent was evaporated to give the crude product, which was purified by flash chromatography (CH_2Cl_2 to 9:1 CH_2Cl_2/MeOH). This gave Ac-L-Arg(Pmc)-$NHCH_3$ as a yellow gum (61 mg, 42%). R_f 0.32 (9:1 CH_2Cl_2/MeOH); δ_H (270 MHz, $CDCl_3$): 1.27 (6H, s, C(CH_3)$_2$) 1.50-1.79 (6H, m, (CH_3)$_2$C$CH2$ + γ CH_2 + β CH_2)1.95 (3H, s, CH_3O) 2.06 (3H, S, ArCH_3) 2.49-2.72 (8H, m, 2 x ArCH_3, CH_2) 2.85, 2.93 (1.5H each, s, $NHCH_3$ rotomers) 3.09-3.29 (NH) 4.37-4.48 (1H, m, α CH) 6.36 (2H, br, NH_2) 7.25 (1H, m, NAcNH) 7.37 (1H, m, CON$H$$CH_3$); δ_C (101 MHz, $CDCl_3$): 12.23 (ArCH$_3$), 17.58 (ArCH$_3$), 18.64 (ArCH$_3$), 21.50 (CH$_2$), 23.07 (NAcCH_3), 25.50 ((CH_3)$_2$CCH$_2$$CH_2$), 26.26 ($CH_3$)$_2$), 26.83 (($CH_3$)$_2$), 29.81 (β CH$_2$), 32.90 ((CH_3)$_2CCH_2$), 40.40 (δ CH$_2$), 53.55 (α CH), 73.80 (C=N), 118.15 (C-CH$_3$), 124.25 (C(CH_2CH_2C(CH_3)$_2$), 133.04 (C-S), 134.86 (C-CH_3), 135.42 (C-CH_3), 153.78 (C-

$OC(CH_3)_2$), 156.59 (C-NH_2), 171.24 (NAc*C*=O), 172.95 (*C*=O($NHCH_3$); HRMS(ES+) found 496.2572 $[M+H]^+$, $C_{23}H_{38}N_5O_5S$ requires 496.2588.

Acylation:

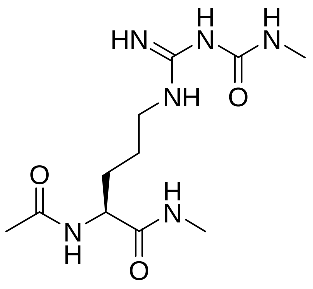

A stirred solution of Ac-Arg(Pmc)-$NHCH_3$ (0.031 g, 0.063 mmol) in CH_2Cl_2 (1 mL) was treated with a solution of 1:1 TFA-CH_2Cl_2 (0.3 mL) and stirred for 2 h at 30 °C. The solvent was evaporated and the residue was dried thoroughly *in vacuo*, and then partitioned between CH_2Cl_2 (2 mL) and water (2 mL). The organic extract was dried ($MgSO_4$) and the solvent was evaporated to give crude Ac-L-Arg-$NHCH_3$ as the TFA salt (15 mg), which was immediately neutralised with pre-washed Dowex hydroxide resin (200 mg) in MeOH (2 mL) for 1 h. The resin was filtered off and the solvent was evaporated to give Ac-Arg-$NHCH_3$ as the free base (7.5 mg, 0.033 mmol assumed). A solution of this material in dry DMF (0.35 mL) was cooled to 0 °C and was treated with a 0.31M solution of methylisocyanate in DMF (0.06 mL, 1.8 equiv). The reaction mixture was allowed to warm to room temperature and was stirred for 18 h, then the solvent was evaporated and the crude product was analysed by mass spectrometry, which indicated partial conversion to **(xxx)** had occurred. The acylation reaction was repeated exactly as before, and the crude product now obtained was purified by RP semi-preparative HPLC (gradient 3) to give **(xxx)** as the trifluoroacetate salt as a yellow solid (2.0 mg, 8% yield). RP-HPLC (analytical, gradient 4, λ = 214 nm): t_R = 8.2 min. **δ_H (400MHz, D_2O):** 1.52-1.59 (2H, m, γ CH_2), 1.65-1.69 (2H, m, β CH_2), 1.91 (3H, s, OCH_3), 2.56 (3H, s, $NHCH_3$), 2.60 (3H, s, $NHCH_3$), 3.25 (2H, t, J=8.8, δ CH_2), 4.06 (1H, t, J=8.4, α CH); **δ_C (101 MHz, D_2O):** 24.12 (γ CH_2), 25.81 ($NHCH_3$), 26.18 ($NHCH_3$), 28.31 (β CH_2), 40.70 (δ CH_2), 53.61

(α CH), 174.38 (C=O), 174.51 (C=O); HRMS(ES+) found 287.1812 $[M+H]^+$, $C_{11}H_{23}N_6O_3$ requires 287.1826.

Guanylurea

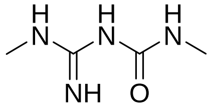

A stirred solution of 1-methylguanidine hydrochloride (0.10 g, 0.91 mmol) in MeOH (6 mL) was treated with pre-washed Dowex hydroxide resin (0.95 g) for 1 h. The resin was filtered off and the solvent was evaporated. A solution of the resulting methylguanidine free base (81 mg, 0.91 mmol assumed) in DMF (0.50 mL) was cooled to 0°C and treated with methylisocyanate (44 μL, 0.74 mmol), then the reaction mixture was allowed to warm to room temperature and was stirred for 16 h. The solvent was evaporated and the crude product was purified by preparative HPLC (gradient 5) to give **(guanylurea)** as the trifluoroacetate salt as a white solid (55 mg, 25%). RP-HPLC (analytical, gradient 6, λ = 214 nm): t_R = 3.2 min**.** δ_H (270MHz, D_2O): 2.67 (3H, s, CH_3), 2.84 (3H, s, CH_3), 2.86 (1H, s, NH), 3.14 (1H, s, NH), 3.20 (1H, s, NH), 3.36 (1H, s, C=NH); δ_C (67.5 MHz, D_2O): 25.99 (CH_3), 27.45 (CH_3), 114.28 (C=NH), 170.16 (C=O)**;** HRMS(ES+) found 131.0932 $[M+H]^+$, $C_4H_{11}N_4O$ requires 131.0927.
